# OCT-measured plaque free wall angle is indicative for plaque burden: overcoming the main limitation of OCT?

**DOI:** 10.1007/s10554-016-0940-y

**Published:** 2016-07-20

**Authors:** Ayla Hoogendoorn, Muthukaruppan Gnanadesigan, Guillaume Zahnd, Nienke S. van Ditzhuijzen, Johan C. H. Schuurbiers, Gijs van Soest, Evelyn Regar, Jolanda J. Wentzel

**Affiliations:** 1 Biomechanics Laboratory, Department of Cardiology, Biomedical Engineering, EE2334b, Erasmus MC, PO Box 2040, 3000 CA Rotterdam, The Netherlands; 2 Biomedical Imaging Group Rotterdam, Departments of Radiology & Nuclear Medicine and Medical Informatics, Erasmus MC, Rotterdam, The Netherlands; 3 Interventional Cardiology, Department of Cardiology, Erasmus MC, Rotterdam, The Netherlands

**Keywords:** Optical coherence tomography, Intravascular ultrasound, Plaque free wall, Plaque burden, Stent-landing zone

## Abstract

The aim of this study was to investigate the relationship between the plaque free wall (PFW) measured by optical coherence tomography (OCT) and the plaque burden (PB) measured by intravascular ultrasound (IVUS). We hypothesize that measurement of the PFW could help to estimate the PB, thereby overcoming the limited ability of OCT to visualize the external elastic membrane in the presence of plaque. This could enable selection of the optimal stent-landing zone by OCT, which is traditionally defined by IVUS as a region with a PB < 40 %. PB (IVUS) and PFW angle (OCT and IVUS) were measured in 18 matched IVUS and OCT pullbacks acquired in the same coronary artery. We determined the relationship between OCT measured PFW (PFW_OCT_) and IVUS PB (PB_IVUS_) by non-linear regression analysis. An ROC-curve analysis was used to determine the optimal cut-off value of PFW angle for the detection of PB < 40 %. Sensitivity, specificity, positive predictive value (PPV) and negative predictive value (NPV) were calculated. There is a significant correlation between PFW_OCT_ and PB_IVUS_ (r^2^ = 0.59). The optimal cut-off value of the PFW_OCT_ for the prediction of a PB_IVUS_ < 40 % is ≥220° with a PPV of 78 % and an NPV of 84 %. This study shows that PFW_OCT_ can be considered as a surrogate marker for PB_IVUS_, which is currently a common criterion to select an optimal stent-landing zone.

## Introduction

In recent years, intravascular optical coherence tomography (OCT) has emerged as imaging technique for guiding percutaneous coronary intervention (PCI). OCT offers a very high spatial and lateral resolution compared to intravascular ultrasound (IVUS). However, this comes at the expense of penetration depth into the tissue [1]. Based on IVUS studies [2, 3], the plaque burden (PB) is a leading criterion for a stent-landing zone, where areas with PB < 40 % [4] are considered optimal. The ability of OCT to visualize PB is limited by optical attenuation: the signal decreases with depth in tissue. Either a thick layer of fibrous (low-attenuation) plaque or the presence of superficial lipids or dense macrophage accumulations (high-attenuation) [5], may obscure the visibility of the media behind the plaque. This potentially limits the use of OCT to guide stent implantation when it comes to the selection of the optimal stent landing zones within the target artery. More recently, in addition to low plaque burden, the absence of lipid/necrotic core within the landing zone has been advocated as a decision criterion which can very reliably be identified by OCT, in contrast to IVUS [6].

In a previous study [7], we demonstrated a strong inverse linear relationship between PB and the plaque free wall (PFW) angle using IVUS imaging (PB_IVUS_ and PFW_IVUS_). In the present study we hypothesize that the angle of the PFW measured in OCT (PFW_OCT_) can likewise reflect the PB. Second, we postulate that regions with a PB < 40 % can be selected for finding an optimal stent-landing zone. When the PFW angle is small (<180°), it might be instantly clear that disease is present with a PB > 40 %, thus forming a suboptimal stent-landing zone. Similarly, sections with a large PFW angle >270° can be easily and reliably identified by OCT as optimal stent-landing zones with a PB < 40 %. However, in the intermediate region between 180°–270° PFW, the interpretation might be more ambiguous. The aim of this study was to investigate the relationship between PFW_OCT_ and PB_IVUS_ and establish the utility (expressed in predictive values for PB < 40 %) of this relationship to detect PB < 40 % for selection of the optimal stent landing zone by OCT.

## Materials and methods

### Study population

To study the predictive value of the PFW_OCT_ for determination of the PB, we performed a retrospective observational study. NIRS-IVUS (TVC, InfraRedx, Burlington, Massachusetts, USA) and OCT (St. Jude Medical Inc., St. Paul, MN, USA) pullbacks of the same ROI were used from 18 left anterior descending (LAD) coronary arteries of 18 patients with stable or unstable coronary syndrome included in the OC3T study (Erasmus MC, Rotterdam).

The NIRS-IVUS images were acquired using a commercially available hybrid optical/ultrasound catheter with an automated pullback (pullback speed 0.5 mm/s and 16 frames per second). The OCT images were obtained with C7-XR/Illumien and Dragonfly catheter. Automated OCT pullback (pullback speed 20 mm/s) was performed during simultaneous iso-osmolar X-ray contrast medium (Visipaque 320, GE Healthcare, Buckinghamshire, U.K.) delivery through the guide catheter, using a power injector (Medrad ProVis, Bayer HealthCare LLC, NJ, USA; typical flush rate 3.0 ml/s).

### Analysis

The OCT and IVUS pullbacks were matched by overlaying the frames with the same side branches. The remainder of the frames was linearly interpolated in between the side branches. Matched NIRS-IVUS and OCT images were analyzed every millimeter over a ROI of the most proximal 20 mm of a pullback. On the IVUS images, the external elastic lamina (vessel area = VA) and lumen area (LA) contours were drawn using QCU-CMS software (version 4.69, Leiden University Medical Centre, LKEB, Division of Image Processing). These contours were used for the calculation of the plaque area (PA = VA − LA) and subsequently the PB (PA/VA × 100 %). PFW angle was defined from the center of the lumen as the arc of the cross-section with a visible, healthy wall having an intima-media thickness of less than 0.5 mm in both imaging modalities. In total, 360 frames were analyzed on both the IVUS and OCT images. The frame data were averaged over 3 mm sections to increase robustness and to compensate for imprecise matching. Images that showed a side branch either on IVUS or OCT, with the wall out of view or a bad flush were excluded. This resulted in 106 matched IVUS and OCT 3 mm sections suitable for analysis. A total of 10 out of the 18 pullbacks of OCT and IVUS were analyzed by two independent expert observers for assessment of the reproducibility.

### Statistics

Statistical analysis was performed using SPSS software (version 21.0, SPSS Inc., Chicago, IL, USA). Findings were regarded significant when p < 0.05. The reproducibility of all PFW and PB measurements was assessed by performing an inter-observer agreement analysis to calculate the intraclass correlation coefficient.

Both a linear and a non-linear model were used to assess the relation between PFW_IVUS/OCT_ and PB_IVUS_. The non-linear model was defined as follows:

$$P{{B}_{IVUS}}=\text{a}\times PFW+\text{b}\ \text{for}\ PFW\le \frac{\left( \text{e}-\text{b} \right)}{\left( \text{a}-\text{d} \right)};$$

$$P{{B}_{IVUS}}=\text{d}\times PFW+\text{e}\ \text{for}\ PFW>\frac{\left( \text{e}-\text{b} \right)}{\left( \text{a}-\text{d} \right)};$$

and was computed for both PFW_OCT_ and PFW_IVUS_. For the non-linear regression analysis, the following starting parameters were chosen: a = 0; b = 55; d = −0.2; e = 100. We denote the crossover value between both linear regimes as PFW_C_ = (e − b)/(a − d), resulting from the regression.

To test whether the slope of the relationship between PFW_OCT_ vs PB_IVUS_ and PFW_IVUS_ vs PB_IVUS_ was similar, implying that with PFW_OCT_ and PFW_IVUS_ the same PB is predicted, a student’s *t* test for two independent samples was used. An ROC-curve analysis was performed to find the optimal cut-off value of PFW_OCT_ to predict a PB_IVUS_ < 40 %. Based on the optimal cut-off value, the sensitivity, specificity, positive predictive value (PPV) and negative predictive value (NPV) of PFW_OCT_ for prediction of PB_IVUS_ < 40 % were calculated.

## Results

### Relation between PFW and PB in IVUS

**Fig. 1:**
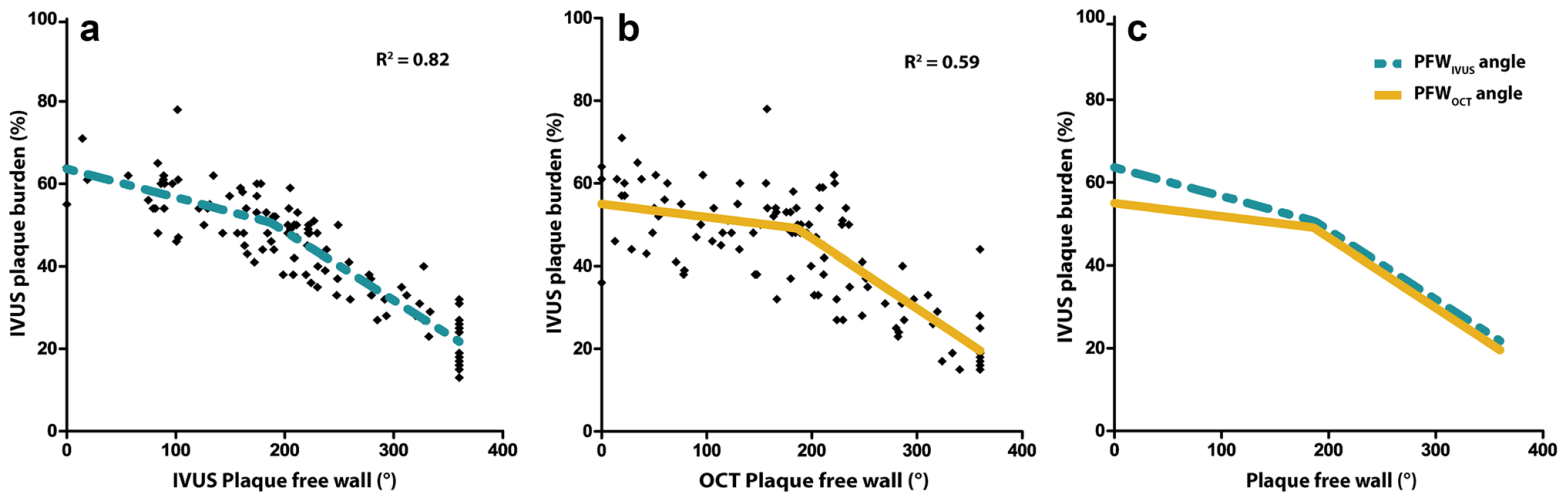
Relationship between the plaque free wall (PFW) angle and plaque burden (PB). **a** The PFW_IVUS_ shows a strong inverse, non-linear correlation with the PB_IVUS_ with a crossover point PFW_C_ = 182° PFW. Final parameters: a = −0.069; b = 63.59; d = −0.163; e = 80.74. **b** A similar relationship is seen for the PFW_OCT_ angle with the PB_IVUS_ with a crossover point PFW_C_ = 186°. Final parameters: a = −0.032; b = 54.98; d = −0.169; e = 80.59. **c** Overlay of regression lines of (**a**) and (**b**). PFW_OCT_ and PFW_IVUS_ perform equally well in predicting PB in the region of PFW angle >186°. In the more diseased regions, the predictive value of the PFW_OCT_ angle is reduced compared to PFW_IVUS_

PFW_IVUS_ and PB_IVUS_ were significantly, inversely related (r^2^ = 0.78) which confirmed the findings of our previous publication [7]. However, a closer fit to the model was found by applying a non-linear regression line (r^2^ = 0.82) (Fig. 1a), with PFW_C_ = 182°.

### Predictive value of PFW_OCT_

To assess whether the PFW_OCT_ could serve as a surrogate marker for PB_IVUS_, the PFW_OCT_ values were plotted against the PB_IVUS_ values. Again, a non-linear regression relationship proved significant (r^2^ = 0.59) (Fig. 1b) with PFW_C_ = 186°. Interestingly, both IVUS and OCT show the same slope (student’s *t* test for inequality of slopes; p = NS) to predict PB for PFW ≥ 186°. For PFW < 186°, PFW_OCT_ had no clear relationship with the PB, contrasting the PFW_IVUS_ which still showed a weak relationship with the PB (Fig. 1c).

**Fig. 2:**
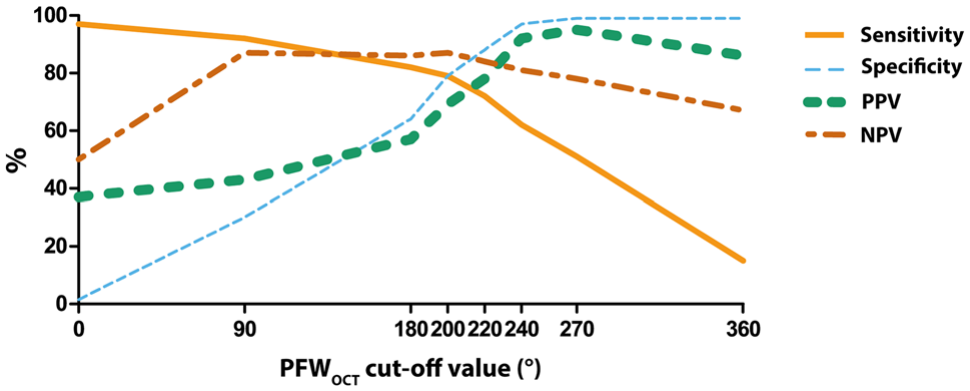
Sensitivity, specificity, PPV and NPV values for different PFW_OCT_ angle cut-off points to predict a PB < 40 %. The optimal cut-off point is set at 220° PFW_OCT_

**Fig. 3:**
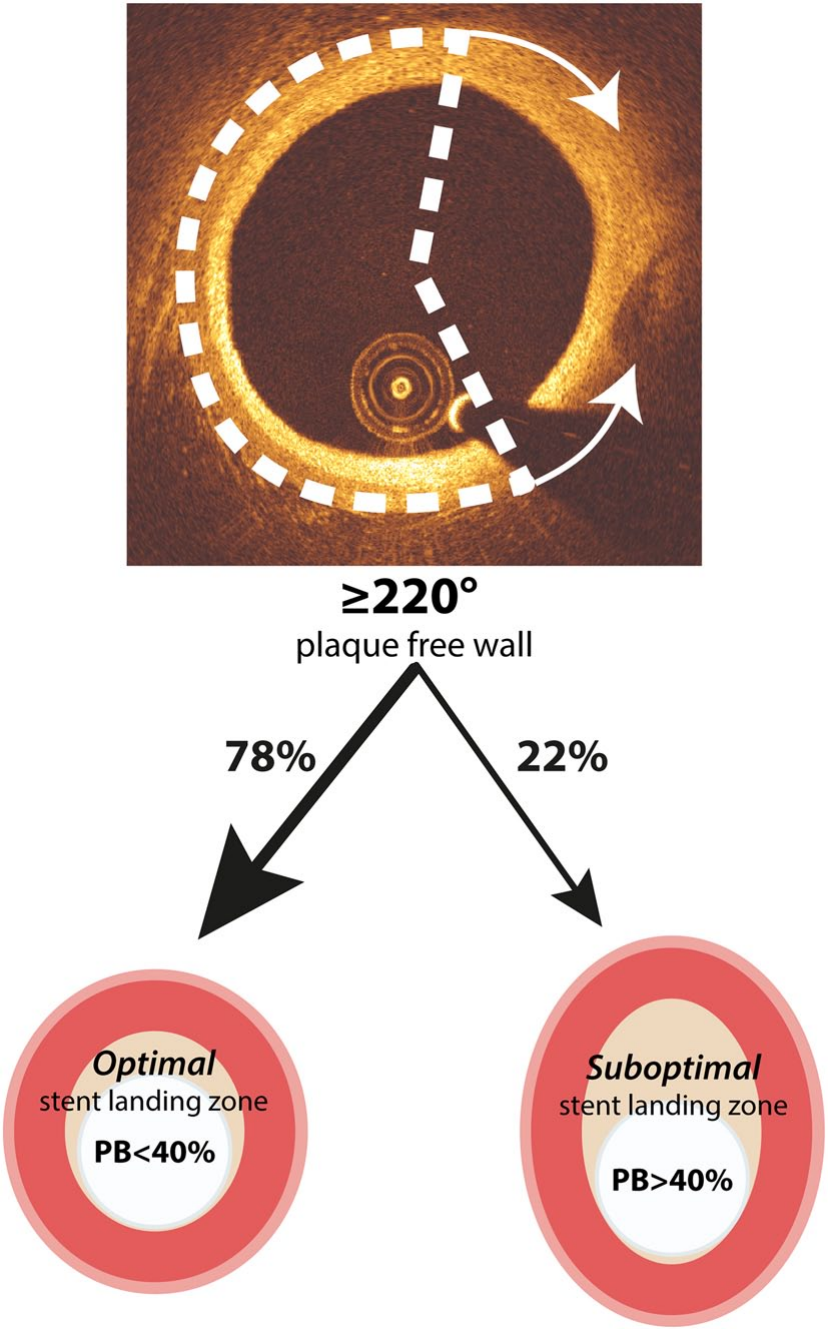
Clinical application of PFW_OCT_ angle detection. When a PFW_OCT_ of ≥220° is detected, in 78 % of the cases this indicates the presence of a PB < 40 %, forming an optimal stent landing zone

To find the optimal cut-off value of the PFW_OCT_ to predict a PB_IVUS_ < 40 %, an ROC-curve analysis was performed. Optimization of sensitivity, specificity, PPV and NPV resulted in a PFW_OCT_ cut-off value of ≥220° (Fig. 2). With this cut-off value, an optimal stent-landing zone (PB < 40 %) could be predicted correctly in 78 % of the cases (PPV) (Fig. 3). Just as important, the NPV was 84 %, meaning that if the PFW angle is <220°, PB > 40 % and thus forming a suboptimal stent-landing zone in 84 % of the cases. This high negative predictive value will largely prevent stenting too far into a health vessel region (PB < 40 %).

As an angle of 220 degrees might be difficult to judge by simple eyeballing in the cathlab, we investigated the predictive value of a PFW > 180 degrees for PB < 40 %. Despite the strong relationship between PFW_OCT_ and PB_IVUS_, regions with a PFW angle >180° can be regarded as an optimal stent landing zone with a PB < 40 % in only 57 % of the cases. So strikingly, in 43 % of the cases the disease is more severe with a PB > 40 %, even with only half of the circumference being occupied by plaque.

### Reproducibility

Inter-observer agreement analyses of the PFW measurements were performed in 189 OCT and 177 IVUS frames and showed high intraclass correlation coefficients of respectively 0.956 (95 % CI 0.941–0.967) and 0.912 (95 % CI 0.884–0.934). The intraclass correlation coefficient of the PB_IVUS_ measurements was equally high at 0.886 (95 % CI 0.847–0.915).

## Discussion

This study shows for the first time that PFW_OCT_ can be considered as a surrogate marker for PB, which is currently a common criterion to determine stent-landing zone. In regions with a PFW angle >186°, the PFW_OCT_ has the same predictive value compared to PFW_IVUS_. The optimized cut-off value to predict optimal stent landing zone (PB < 40 %) is ≥220° PFW_OCT_.

There is a precarious balance between adequate lesion coverage and using too long stents. When the edge of the stent lands in a plaque area, it poses a risk on plaque disruption and edge dissections on a short-term and in-stent restenosis on a long-term [8]. On the other hand, placing longer stents might prove technically more challenging and might increase the risk on in-stent restenosis and stent thrombosis, even in drug eluting stents [9–11]. For this reason, determining an optimal stent-landing zone is critical for the prevention of future stent-related adverse events.

An optimal stent-landing zone is a region without lipids and a PB < 40 %. In contrast to IVUS, OCT is capable of detecting lipids, but the limited penetration depth hampers PB measurements. OCT can however reliably detect the healthy vessel wall. Therefore, we aimed to investigate if the presence of a normal wall is helpful to predict PB by OCT. This study showed that a measurement of the PFW angle provides a reliable estimate of the PB, without the need to fully visualize the outer wall of the vessel. Together with the fact that OCT is one of the most reliable techniques to structurally assess the presence of a lipid rich plaque, determining the stent-landing zone by OCT becomes even more feasible [12, 13].

The detection of the PFW in OCT can be influenced by the composition of the intima. Plaque components like a lipid/necrotic core and sites of inflammatory activity cause high attenuation and may obscure the vessel wall layers, even in a relatively thin intima. Plaque-free regions (IMT < 0.5 mm) with a superficial layer of macrophages or foam cells (intimal xanthoma) can appear as regions with plaque, since the healthy 3-layered structure of the vessel is not visible. This leads to underestimation of the PFW angle and thus overestimation of the plaque burden. The confounding effect of vessel wall composition may explain why the association between PFW_OCT_ and PB almost vanishes in the more diseased sections. We observe a similar trend in the relation between PFW_IVUS_ and PB, although the detection of the PFW by IVUS is usually not affected by attenuation-inducing plaque components. This might indicate a possible biological phenomenon. In the initial stages of plaque growth, the asymmetric plaque growth results in a simultaneously decreasing PFW angle and increasing PB. Apparently in later stages, the plaque growth becomes more symmetrical, decreasing the PFW angle with only a limited increase in PB and thus weakening the relationship. This explanation should be supported by serial imaging studies.

PCI procedures are always guided by angiography to determine catheter position and lumen narrowing. Currently, it is challenging to identify segments seen on OCT with the corresponding location on angiography which could lead to wrong sizing and positioning of the stent. The recently introduced on-line co-registration of OCT and angiography [14] could greatly increase the utility of OCT and thereby the application of our findings in the clinical work flow. Furthermore, the most predictive cut-off angle of 220° PFW is hard to determine by eye. This might limit direct clinical application of our findings. New software for the automatic detection of the three wall layers and the determination of the PFW angle is under development in our university.

### Limitations

Some limitations in our study should be taken into account. Despite the use of side branches for optimal matching of OCT and IVUS pullbacks, errors in matching can occur due to intrinsic differences between the two techniques. These include, amongst others, pullback speed, lateral resolution and frame rate. To minimize the impact of these mismatches on the accuracy of the results, the measurements were averaged over a region of 3 mm, which is a larger than the maximum expected longitudinal mismatch.

Only a limited number of matched pullbacks were available for analysis. Since we analyzed the IVUS images per cross-section and not longitudinally, we regarded the seven data points per pull-back as separate observations. We do not expect that our conclusion would be much different with a larger dataset. However, these results should still be interpreted as being a proof-of-principle study.

### Conclusion

In conclusion, this study shows that PFW_OCT_ is a good predictor for the PB in the regions with a PFW angle >186° and gives more insight in the general interpretation of PFW_OCT_ angles. After further clinical validation, this new PB estimation based on OCT could help to identify the optimal landing zone.
